# Depression screening and advisory service provided by community pharmacist for depressive students in university

**DOI:** 10.1186/s40064-015-1259-1

**Published:** 2015-09-02

**Authors:** Wiraphol Phimarn, Pongsatorn Kaewphila, Siritree Suttajit, Kritsanee Saramunee

**Affiliations:** Social Pharmacy Research Unit, Faculty of Pharmacy, Mahasarakham University, Kantharawichai, Maha Sarakham, Thailand; Clinical Pharmacy Research Unit, Faculty of Pharmacy, Mahasarakham University, Kantharawichai, Maha Sarakham, Thailand; Pharmaceutical Care Department, Faculty of Pharmacy, Chiangmai University, Chiangmai, Thailand

## Abstract

**Background:**

Depressive symptom among adolescent is prevalent but advisory service for this symptom is limited, particularly in university.

**Objectives:**

(1) To identify depressive students in health science faculties, (2) To evaluate the consequence of depression advisory service by community pharmacist, compared between a group counseling and an individual one.

**Methods:**

A two-phase study was designed—a cross-sectional study followed by an experimental study. Health science students were screened by CES-D questionnaire. The prevalence and predictors of depressed mood were determined. Depressive students were then invited to the experimental study. Participants were assigned into 2 groups, by stratified random sampling, and followed up for 16 weeks. Group 1 received a group counselling, group 2 received an individual counselling from a trained pharmacist. Outcomes measured were the CES-D score and quality of life.

**Results:**

The prevalence of depressed mood students was 13.7 % (195/1421). Students in year 2nd and year 3rd, nursing and medicine students, and GPA were strongly associated with the CES-D score (*P* < 0.05). Sixty-eight depressive students were assigned into the experiment. The CES-D scores of both groups were significantly reduced from the baseline (*P* < 0.001). The post-test score of group 2 was lower than group 1 (17.7 ± 4.5 vs 20.1 ± 4.6, *P* = 0.038). At week 16, both counselling types significantly increased mean score of physical health (*P* < 0.001) whereas score of mental health was increased significantly only by the individual counselling, from 37.9 ± 9.9 to 43.1 ± 8.4 (*P* = 0.036).

**Conclusions:**

Depressive symptom among health science students is considerably high. Year of study, faculty and GPA are significant predictors of this disorder. Trained community pharmacists can effectively screen and provide advisory service. Individual counseling is more effective than using group advice.

## Background

Depression is a common mental disorder that presents with depressed mood, loss of interest or pleasure, feelings of guilt or low self-esteem, disturbed sleep or appetite, low energy, and poor concentration. Clearly, depression has a significant impact on an individual’s ability to perform life activities and also leads to increased risk of self-injury, attempting or committing suicide (White et al. 2012). The World Health Organization (WHO) stated that depression is one of the major health problems. The global health survey found that approximately 1 in 20 persons reported having depression in the past year; this depressive symptom often begins at young age (World Federation for Mental Health 2012). Prevalence of depression among adolescents is high. A previous study estimated prevalence of depression or anxiety among undergraduate students was 15.6 % (Eisenberg et al. 2007), while the more recent one found 9–13 % (Richards et al. 2014). Students normally experienced their first episode in college (Mowbray et al. 2006). However, currently, many depressive students have not yet been diagnosed (Ovuga et al. 2005). If those were identified and provided appropriate health service, serious consequences of depression and suicide would be reduced (Wang et al. 2007).

Mahasarakham University (MSU), located in Northeastern Thailand, is an educational institute that provides various undergraduate programs. Depression is also problematic as found in other universities. Previous studies indicate prevalence of depression as 18.7 % (Wisethorn 2008) and 14.6 % (Wonghirunkul et al. 2010) for pharmacy and social science students, respectively. Nonetheless, the study among health science students has never been conducted, but is of importance, because of expected high stress. A study in Malaysia revealed that health science students reported having moderate to high stress level due to academic requirements (Othman et al. 2013).

A service for mental problems called ‘hotline’, a telephone counseling service, is available for MSU students. This service has been provided to students whose mental health is poor. Moreover, the mental health advice is also provided by a psychiatrist via the university medical center. But, students must contact these places by themselves to seek advice, thus limiting accessibility. There is a shortage of health professionals specializing in psychiatry in Thailand. The number of psychiatrists has increased slowly in the past decade, 351 in 1999 to 750 in 2013 (Department of Mental Health 2013). Expanding mental health service is, therefore, difficult. Experimental studies demonstrated that pharmacist intervention improved adherence of patients taking antidepressant medication (Capoccia et al. 2004) and tended to be cost-effective (Rubio-Valera et al. 2012). One experimental study in Thailand demonstrated that pharmacist counselling could help reduce depressed mood in humanistic and social science students (Wonghirunkul et al. 2010). Pharmacists had a positive attitude on the role for mental health advice, but were not confident to perform such a task because of a lack of training (Scheerder et al. 2008). However, the result of an Australian survey showed high mental health literacy among pharmacists (O’Reilly et al. 2010). This evidence suggests that community pharmacists have potential to be involved in care for depressive symptom. To lessen the accessibility gap, a community pharmacy located on the MSU campus sought to develop a depression screening system and advisory service to help depressive students, especially those studying in health sciences. A two-phase study was designed. Phase I was a cross-sectional study to determine the prevalence of depressive symptom among health science students and to examine statistical relationships between depressive symptom level and student demographic factors. Phase II was an experimental study to examine and compare outcomes between community-pharmacist-provided group counselling and individual counselling of students with depressive symptoms. This study will enable us to propose a screening system and appropriate service to be implemented in the university health system.

## Methods

### Phase I: a cross-sectional study

#### Instruments

##### Depressive symptom screening questionnaire

A standard screening questionnaire by the Centre for Epidemiologic Studies-Depression Scale (CES-D) Thai version was used since it is widely employed for screening adolescents having depressed mood. The questionnaire was validated showing high internal consistency with Cronbach’s alpha coefficients 0.86. Sensitivity, specificity and accuracy of this version were 72, 85 and 82 %, respectively (Trangkasombat et al. 1997). The questionnaire contains 20 four-scale questions (rarely = 0, occasionally = 1, some = 2, most = 3). The total score was computed by summation of the score given for each question, thus ranging from 0 to 60. It is recommended that individuals with the CES-D score ≥22 were potentially having depressive mood, so-called ‘depressive student’ (Trangkasombat et al. 1997). A form to record the student profile was also enclosed as we wished to identify demographic factors relating to depressed mood. Demographic factors were gender, age, faculty and year of study which were correlated to depressive symptom stated previously (Othman et al. 2013; Shamsuddin et al. 2013). Grade Point Average (GPA), used as a key indicator for study performance, was also recorded because academic requirement was proved to be a major stressor for students (Othman et al. 2013). Name and telephone number were requested only to further contact the participant regarding the experimental study.

##### Suicidal screening questionnaire

Participants with CES-D score ≥22 were asked to complete a suicidal screening questionnaire because depressive symptom may lead to suicide (Yongthong et al. 2013). This questionnaire was developed by the Department of Mental Health, Ministry of Public Health of Thailand (2005) (Ministry of Public Health 2014). It was specific to assess suicidal ideation of participants in the past 2 weeks. This questionnaire contains 10 dichotomous questions (yes/no). The answer of ‘yes’ to at least one question means that participant was at risk of suicide. In this step, all participants with suicidal ideation were referred to the hospital.

##### Screening procedure

Depressive symptom screening procedure was conducted in July 2010. All students from four health science faculties of MSU (nursing, medicine, pharmacy and public health) were invited to self-complete the CES-D questionnaire. Those with CES-D score ≥22 were asked to complete the suicidal screening subsequently. If they were already diagnosed with depression, they were then excluded from the study. If they were at risk of suicide, they were then referred to the hospital.

##### Statistical analysis

Multiple linear regression was performed to identify potential predictors of depressive symptom by using CES-D score as a dependent variable. Independent variables including gender, age, faculty, year of study and GPA were entered in the analysis by stepwise method. Stepping method criteria used *P* value of F which was set at 0.05 for entry and 0.10 for removal.

### Phase II: experimental study

#### Study design

A randomized controlled trial was conducted at a community pharmacy located on the university campus. The trial was undertaken from August to December of 2010.

#### Participants

The target population was depressive students, found in phase I. Optimal sample size was calculated using the formula below. Parameters used for calculation were based on statistical standard practice (Z_α_ = 1.96) and previous research, which reported the change of depressed outcomes due to pharmacist’s intervention (σ^2^ = 6.22, E = 2.26) (Wisethorn 2008). This indicated that the appropriate number of participants should be at least 25 in each group, but in this study that number was increased by 30 % in order to account for loss of follow up. Therefore, the appropriate number of participants was 32. All depressive students were invited to the study; however, 68 enrolled into the trial eventually.

$${\text{n}} = {\text{NZ}}^{ 2}_{\alpha } \sigma^{ 2} /({\text{NE}}^{ 2} + {\text{ Z}}^{ 2} \sigma^{ 2} )$$

#### Randomization

Participants were assigned to either group 1 or group 2 by stratified random sampling. To ensure demographic parity of the two groups, they were allocated based on faculty, year of study and gender.

#### Intervention

**Fig. 1:**
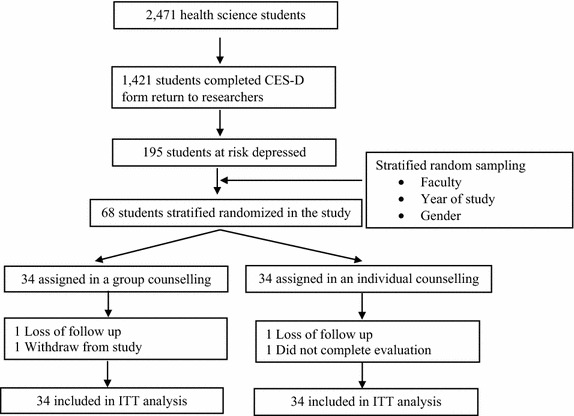
Study flow chart

Two community pharmacists were trained by the psychiatric pharmacist for two-weeks at a neurological hospital prior to the study. They practiced giving advice to a mock client. A flowchart describing the interventions used in the experimental study is shown as Fig. 1.

Participants in group 1 (n = 34) received group counselling regarding depressive symptom by a trained community pharmacist. They were scheduled to visit the community pharmacy on a pre-determined day that enabled them to attend the group counselling together. The group session lasted approximately 1 h, covering information about definition and cause of depressive symptom, risk factors, signs, and types of depression, symptoms, treatments, and self-managements. The group counseling sessions were provided at week 0, 4, 8 and 16. Outcomes were measured as described below.

Participants in group 2 (n = 34) received individual counselling from one of the two trained pharmacists. Participants were scheduled to visit the community pharmacy. At week 0, the depression management handbook was provided to all participants for self-study. The handbook developed by a research team was comprised of three parts (a) an overview about depressive symptom including definition, causes, risk factors of depressive symptom and self-managements, (b) a patient profile recording personal information and clinical symptoms, and (c) a form for patients to record their daily activities to relieve depressive symptoms. Participants received individual counseling at week 0, 4, 8, 16 from the same pharmacist. The counseling sessions lasted approximately 1 h and focused on finding the cause of depressive symptom and method to relieve depressed mood. Outcomes were measured as described below.

### Outcome measurement

#### Depressed mood level

Depressed mood level was a key outcome measured by the CES-D questionnaire. Assessment was carried out at week 0 before the counselling started and at week 16 after the counselling was completed. Mean CES-D score was calculated for both groups.

#### Quality of life (QOL)

Previous studies reported that mental health problems, as in the case of other chronic diseases, generally affects quality-of-life (QOL) (Rapaport et al. 2005; Kanjanasilp et al. 2009), and pharmacist intervention was shown to impact QOL of primary care patients with depression (Rubio-Valera et al. 2013). This study, therefore, evaluated and compared QOL before and after the pharmacist intervention. The Short Form (36) Health Survey (SF-36) Thai version was used to measure QOL. Lim et al. (2008) validated this instrument thoroughly and found good reliability of each domain test, presenting the Cronbach’s alpha coefficient greater than 0.70. SF-36 consisted of 36 items clustered to measure eight domains of QOL: physical functioning (PF), role-physical (RP), bodily pain (BP), general health (GH), vitality (VT), social functioning (SF), role-emotional (RE), and mental health (MH). Details of scales and scoring were explained by Lim et al. (2008). We calculated the mean score for physical health by the summed score of PF, RP, PB and GH divided by four. Similarly, we then calculated the mean score for mental health by the summed score of VT, SF, RE and MH divided by four.

#### Statistical analysis

Statistical analysis was performed using the statistical package for the social science (SPSS) version 16. All analyses used the intention-to-treat (ITT) approach with a use of sequential hot deck imputation to manage missing data. Descriptive statistics to report results included mean, standard deviation, and percentage as appropriate. Statistical differences within the same group were tested using paired t-test while an independent t-test was used for testing the differences between two groups. The level of statistical significance was determined to be 0.05 or less.

### Ethical consideration

The study protocol was approved by Mahasarakham University Ethics Committee. Written consent form was obtained. Information obtained from participants was anonymous and kept confidential.

## Results

### Phase I

#### Prevalence and predictors of depressive symptom

A total of 2471 health science students were invited. Of those, 1421 completed the CES-D questionnaire. Public health school had the lowest response rate at 48.7 % while the highest rate was from pharmacy school at 77.5 %, giving overall response rate of 57.5 %. The majority of respondents was female (79.5 %) and age range 18–20 years (62.1 %).

The screening found 195 students had CES-D score equal to or greater than 22, indicating the prevalence of depressive symptom among health science students of 13.7 %. As shown in Table 1, the majority of depressive students was female (77.9 %) and age range was 18–20 years (68.2 %). When comparing between faculties, depressive students were found in high proportion among public health and medicine students, whereas the pharmacy and nursing students were fewer. Students with GPA 2.51–3.00 exhibited depressive symptom most often, 43.6 %. When considering individual items of the CES-D questionnaire, the top three statements that depressive students selected were: I was bothered by things that usually don’t bother me (59.0 %), I had trouble keeping my mind on what I was doing (53.3 %), and I felt depressed (46.7 %).

**Table 1:** Proportion of respondents and depressive students by demographic variables

	Respondents (N = 1421)		Depressive students (N = 195)	
	n	%	n	%
Proportion by gender				
Male	291	20.5	43	22.1
Female	1130	79.5	152	77.9
Proportion by age range				
≤20	882	62.1	133	68.2
21–25	516	36.3	60	30.8
26–30	18	1.3	2	1.0
31–35	4	0.3	0	0.0
More than 35	1	0.1	0	0.0
Proportion by faculty				
Nursing	147	10.3	26	13.3
Medicine	215	15.1	55	28.2
Pharmacy	471	33.1	44	22.6
Public health	588	41.4	70	35.9
Year of study				
Year 1st	343	24.1	47	24.1
Year 2nd	525	36.9	78	40.0
Year 3rd	407	28.6	59	30.3
Year 4th	100	7.0	8	4.1
Year 5th	46	3.2	3	1.5
Proportion by GPA range				
0.00–2.50 (poor)	132	9.3	16	8.2
2.51–3.00 (fair)	410	28.9	85	43.6
3.01–3.50 (good)	586	41.2	61	31.3
3.51–4.00 (excellent)	293	20.6	33	16.9

*GPA* grade point average

Results showed that student year 2nd, student year 3rd, nursing and medicine students, and GPA were strongly associated with the CES-D score (*P* < 0.05) (Table 2).

**Table 2:** Predictors of depression in students identified by multiple linear regression

Variables	β^a^	β^b^	T	*P* value
Year 2nd	12.208	0.087	2.905	0.004
Year 3rd	1.017	0.069	2.266	0.024
Nurse	2.960	0.159	5.975	<0.001
Medicine	1.553	0.771	2.569	0.010
GPA	−1.131	−0.074	−2.798	0.005

^a^ Unstandardized coefficients, ^b^ standardized coefficients, Reference categories for variable faculty and year were pharmacy and year 4th since they showed the least depressive level

### Phase II

#### Participants in the experimental study

Sixty-eight students agreed to take part, 34 in each group. Before the study completion, two participants of both groups were dropped due to loss of follow up, incomplete evaluation and self-withdrawal resulting in 94.1 % of adherence to the intervention. Since we used the ITT approach, all participants were included in the analysis. Demographic characteristics of participants in the two groups were not different at baseline (Table 3).

**Table 3:** Baseline demographic

	Number of sample (N = 68)		*P* value
	Group 1: a group counselling (n = 34)	Group 2: an individual counselling (n = 34)	
Gender			
Female	12	12	1.00^a^
Male	22	22	
Age (mean ± SD)	20.0 ± 0.95	19.9 ± 0.92	0.91^b^
Faculty			
Nursing			
Year 1st	2	2	1.00^a^
Year 2nd	2	2	
Medicine			
Year 1st	3	3	0.99^a^
Year 2nd	6	5	
Year 3rd	3	3	
Pharmacy			
Year 1st	2	3	1.00^a^
Year 2nd	3	3	
Year 3rd	3	3	
Year 4th	2	2	
Year 5th	0	1	
Public health			
Year 1st	2	3	0.99^a^
Year 2nd	3	3	
Year 3rd	2	2	
CES-D score (mean ± SD)	26.30 ± 4.60	25.10 ± 4.20	0.23^b^
Quality of life score (mean ± SD)			
Physical (QOL-P)	43.90 ± 7.80	46.40 ± 8.10	0.22^c^
Mental (QOL-M)	40.0 ± 8.10	37.90 ± 9.90	0.35^b^

Statistical significance was tested by ^a^ Chi square, ^b^ Mann Whitney U test, and ^c^ independent t-test

### Clinical outcomes

#### Depressed mood level

At baseline, there was no statistical significance regarding depressive symptom between the two groups. In addition, the baseline CES-D score did not associate with the post CES-D score tested by linear regression. The results showed that at week 16, of the number of students with the CES-D score <22 indicating normal mood, group 2 was significantly higher than group 1 (27 (79.4 %) vs 18 (52.9 %); *P* = 0.027). Table 4 provides the mean CES-D score of the two groups comparing pre- and post-test. Although both types of counselling were able to decrease the mean CES-D score significantly (*P* < 0.001), the decreased score was greater when counselled individually.

**Table 4:** Mean CES-D score

	CES-D score (mean ± SD)			*P* value
	Week 0	Week 16	∆	
Group 1: a group counselling (n = 34)	26.3 ± 4.6	20.1 ± 4.6	−6.2	<0.001^a,^*
Group 2: an individual counselling (n = 34)	25.1 ± 4.2	17.7 ± 4.5	−7.4	<0.001^a,^*
*P* value	0.230^b^	0.038^b,^*		

Statistical significance was tested by ^a^ paired t-test and ^b^ independent t-test, * *P* value is 0.05 or less

#### Quality of life

Table 5 shows the mean QOL score of the two groups. At baseline, there was no statistical significance regarding QOL score between the two groups. Significant association between pre- and post- QOL score was not found by linear regression. As a result, at week 16, counselling types significantly increased the QOL mean score of physical health (7.8 points for group counselling and 6.7 points for individual counselling) whereas the mean score of mental health was increased significantly only by the individual counselling, from 39.9 ± 9.9 to 43.1 ± 8.4.

**Table 5:** Mean score of quality of life

	QOL score (mean ± SD)			*P* value
	Week 0	Week 16	∆	
Physical domain				
Group 1: a group counselling (n = 32)	43.9 ± 7.8	51.7 ± 6.1	+7.8	<0.001^b,^*
Group 2: an individual counselling (n = 32)	46.4 ± 8.1	53.1 ± 7.7	+6.7	0.003^b,^*
*P* value	0.215^a^	0.419^a^		
Mental domain				
Group 1: a group counselling (n = 32)	40.0 ± 8.1	42.8 ± 8.6	+2.8	0.067^b^
Group 2: an individual counselling (n = 32)	37.9 ± 9.9	43.1 ± 8.4	+5.2	0.036^b,^*
*P* value	0.349^a ^	0.857^a ^		

Statistical significance was tested by ^a^ independent t-test and ^b^ paired t-test, * *P* value is 0.05 or less

## Discussion

This study sought to design a depressive screening system and advisory service in the university health system. The study population was MSU health science students as this group has never been explored. The response rate was 57.6 % while previous studies reported higher response, 65.7–82.7 % (Wisethorn 2008; Schwenk et al. 2010). Prevalence of depressive symptom among MSU health science students was 13.7 %, similar to previous studies. Prevalence of depression among humanistic and social science students was reported at 14.6 % (Wonghirunkul et al. 2010), 24.5 % among pharmacy students (Kanjanasilp et al. 2009), and 16.2 % among nursing students (Vatanasin 2006). Schwenk (2010) reported depression prevalence of medical students in the US as 14.3 %.

Depressive symptom among health science students was predominant in females, in agreement with other studies (Richards et al. 2014; O’Reilly et al. 2010). In terms of factors influencing depressed mood, we found that year of study (year 2nd and 3rd), faculty (medicine and nursing), and GPA strongly affected depressive symptom. Medical and nursing students in year 2nd and 3rd were normally in the rigorous phase of preclinical courses. Schwenk et al. (2010) also found this correlate in American medical study. In addition, Thai medical students generally have the national license examination during study in year 3rd, which possibly causes accumulated stress. For nursing students, high workload from practice in the hospital was a reason of depressed mood. This evidence confirmed the need of mental health service.

Previous studies reported that screening for depression (Pignone et al. 2002) and appropriate counselling significantly improved outcomes (Wonghirunkul et al. 2010; Asarnow et al. 2005): thus, these two procedures were included. Our study showed that the number of students with the CES-D score <22, indicating normal mood, had increased significantly, confirming that appropriate counselling can help alleviate depressive symptom. QOL score measured by SF-36 is a standard tool used widely. As found by others (Wonghirunkul et al. 2010; Kanjanasilp et al. 2009), outcomes of the two interventions in this present study were in agreement. That is, the CES-D score and QOL were improved after interventions were completed. These results, nevertheless, were in contrast with the study by Asarnow et al. (2005), who reported that the service provided by a psychiatry specialist team was more effective than the normal service provided by a general health professional.

Taking care of students is an important role of university staff. Since the number of psychiatric specialists in Thailand is limited, the university should create alternative service to help students with mental health problems. Previous studies indicated that counselling could decrease the level of stress and depression suggesting that a training course for academic staff should be provided to enhance counselling ability (Wonghirunkul et al. 2010; Kanjanasilp et al. 2009; Solanky et al. 2012).

## Strength and limitation

This study was to enhance access to health care of depressed college students. Although, screening for depressive symptom has been conducted before, (Wisethorn 2008; Wonghirunkul et al. 2010; Kanjanasilp et al. 2009) it did not include all health science students, who are at high risk perhaps because of their rigorous academic program (Othman et al. 2013; Solanky et al. 2012); their includion strengthened this study. Outcomes in our study were depressed mood level and QOL measured by standard CES-D and SF-36 questionnaire, ensuring valid and reliable findings. Response rate from pharmacy students was the highest perhaps because the study originated from within the pharmacy school. However, pharmacy students were not a majority of depressive students, so that may not affect the results. In fact, other mental health services were available such as a hotline telephone counselling and psychologists at the university medical center. Participants were not prohibited from seeking those services so these might slightly bias study outcomes. Since, this study did not include the usual care and depressive symptoms can be alleviated naturally. Therefore, the improvement of depressive symptoms and QOL may not be warranted. The sample size used in this study was small and calculated to detect difference in depressive symptoms, which may have limited our ability to detect differences in QOL.

## Conclusion

This study demonstrated high prevalence of depressive symptom among health science students. Year of study, faculty and GPA are significant predictors of this disorder. Trained community pharmacists can effectively screen and provide advisory service leading to improved CES-D score and QOL. Individual counseling seems more effective. Based upon this study we recommend that the university should implement depressive symptom screening to monitor problematic students as well as engage community pharmacy as a partner of university health care.

## References

[CR1] Asarnow J, Jaycox L, Duan N, LaBorde A, Rea M, Murray P (2005). Effectiveness of a quality improvement intervention for adolescent depression in primary care clinics. JAMA.

[CR2] Capoccia KL, Boudreau DM, Blough DK, Ellsworth AJ, Clark DR, Stevens NG (2004). Randomized trial of pharmacist interventions to improve depression care and outcomes in primary care. Am J Heal Pharm.

[CR3] Department of Mental Health (2013) Number of psychiatry in Thailand. In: Department of Mental Health. http://www.dmh.go.th/Plan/Download/ict/mhresource48.pdf. Accessed 3 Aug 2014

[CR4] Eisenberg D, Gollust SE, Golberstein E, Hefner JL (2007). Prevalence and correlates of depression, anxiety, and suicidality among university students. Am J Orthopsychiatry.

[CR5] Kanjanasilp J, Saramunee K, Kongsree S, Suthiraksa S, Chummalee I, Hanrinth R (2009). Effects of counseling for stress and depression in pharmacy students at Mahasarakham University (In Thai). Isan J Pharm Sci.

[CR6] Lim LL, Seubsman SA, Sleigh A (2008). Thai SF-36 health survey: tests of data quality, scaling assumptions, reliability and validity in healthy men and women. Health Qual Life Outcomes.

[CR7] Ministry of public health (2014) CES-D Thai version. In: Ministry of public health. http://www.dmh.moph.go.th/test/cesd/cesd/. Accessed 4 Aug 2014

[CR8] Mowbray CT, Megivern D, Mandiberg JM, Strauss S, Stein CH, Collins K (2006). Campus mental health services: recommendations for change. Am J Orthopsychiatry.

[CR9] O’Reilly CL, Bell JS, Chen TF (2010). Pharmacists’ beliefs about treatments and outcomes of mental disorders: a mental health literacy survey. Aust NZ J Psychiatry.

[CR10] Othman C, Farooqui M, Yusoff M, Adawiyah R (2013). Nature of stress among health science students in a Malaysian University. Procedia Soc Behav Sci.

[CR11] Ovuga E, Boardman J, Wasserman D (2005). The response inventory for stressful life events (RISLE) II: validation of the 36-item version. Afr Health Sci.

[CR12] Pignone MP, Gaynes BN, Rushton JL, Burchell CM, Orleans CT, Mulrow CD (2002). Screening for depression in adults: a summary of the evidence for the U.S. Preventive Services Task Force. Ann Intern Med.

[CR13] Rapaport MH, Clary C, Fayyad R, Endicott J (2005). Quality-of-life impairment in depressive and anxiety disorders. Am J Psychiatry.

[CR14] Richards D, Sanabria A (2014). Point-prevalence of depression and associated risk factors. J Psychol.

[CR15] Rubio-Valera M, Jove A, Hughes C, Guillen-Sola M, Rovira M, Fernandez A (2012). Factors affecting collaboration between general practitioners and community pharmacists: a qualitative study. BMC Health Serv Res.

[CR16] Rubio-Valera M, March Pujol M, Fernández A, Peñarrubia-María MT, Travé P, López Del Hoyo Y, Serrano-Blanco A (2013). Evaluation of a pharmacist intervention on patients initiating pharmacological treatment for depression: a randomized controlled superiority trial. Eur Neuropsychopharmacol.

[CR17] Scheerder G, De Coster I, Van AC (2008). Pharmacists’ role in depression care: a survey of attitudes, current practices, and barriers. Psychiatr Serv.

[CR18] Schwenk TL, Davis L, Wimsatt LA (2010). Depression, stigma, and suicidal ideation in medical students. JAMA.

[CR19] Shamsuddin K, Fadzil F, Ismail WSW, Shah SA, Omar K, Muhammad NA (2013). Correlates of depression, anxiety and stress among Malaysian university students. Asian J Psychiatr.

[CR20] Solanky P, Desai B, Kavishwar A (2012). Study of psychological stress among undergraduate medical students of government medical college, Surat. Int J Medicial Sci Public Heal.

[CR21] Trangkasombat U, Larpboomsup V, Hawanont P (1997). CES-D in adolescents. J Psychiatr Assoc Thail.

[CR22] Vatanasin D (2006) Depression among undergraduate nursing students. Dissertation, Mahidol University

[CR23] Wang PS, Angermeyer M, Borges G, Bruffaerts R, Tat Chiu W, Girolamo G (2007). Delay and failure in treatment seeking after first onset of mental disorders in the World Health Organization’s World Mental Health Survey Initiative. World Psychiatry.

[CR24] White L, Klinner C, Carter S (2012). Consumer perspectives of the Australian Home Medicines Review Program: benefits and barriers. Res Soc Adm Pharm.

[CR25] Wisethorn S (2008) Screening and monitoring depression in Pharmacy student. Dissertation, Mahasarakham University

[CR26] Wonghirunkul W, Phimarn W, Kanjanasilp J (2010). Screening and monitoring depression in humanistic and social science student, Mahasarakham university (In Thai). Thai Pharm Heal Sci J.

[CR27] World Federation for Mental Health (2012) Depression: a global crisis. In: World Health Organization. Available via: http://www.who.int/mental_health/management/depression/wfmh_paper_depression_wmhd_2012.pdf?ua=1. Accessed 3 Aug 2014

[CR28] Yongthong N, Wattanapailin A, Seeherunwong A, Yuttatri P (2013). The effect of a coping enhancing program on depression in breast cancer patients. J Nurs Sci.

